# Acute Carpal Tunnel Syndrome Secondary to Amyloidosis

**DOI:** 10.1155/2019/1610430

**Published:** 2019-11-23

**Authors:** Ömer Serdar Hakyemez, Fatih Arslanoğlu, Murat Birinci, Mehmet Akif Çaçan, Adnan Kara

**Affiliations:** Medipol Mega University Hospital, Istanbul, Turkey

## Abstract

**Introduction:**

ACTS secondary to amyloidosis is a very rare situation in the literature, and here, we present a unique case of ACTS secondary to amyloidosis.

**Case Report:**

A 61-year-old male patient was admitted to our hospital with complaints of numbness in the lateral half of his 1, 2, 3, and 4 fingers of his right hand. These complaints started acutely, and the patient did not have a history of trauma. His clinical examination was suitable for acute carpal tunnel syndrome.

**Discussion:**

Carpal tunnel syndrome, as well as acute carpal tunnel syndrome, may occur based on different causes. ACTS is very rare, especially when it is not caused by a trauma. Here, we presented a unique case of ACTS based on amyloidosis.

**Conclusion:**

It should be kept in mind when ACTS may occur in patients with the diagnosis of amyloidosis.

## 1. Introduction

Amyloidosis is known to be associated with carpal tunnel syndrome (CTS) [1]. Amyloid deposition in the tendon and surrounding tissues further reduces the already low carpal tunnel volume, compressing the median nerve, and causes chronic CTS. However, this is not very expected to occur acutely. Here, we present a unique case of ACTS secondary to amyloidosis.

## 2. Case Report

A 61-year-old male patient was admitted to our hospital with complaints of numbness in the lateral half of the 1, 2, 3, and 4 fingers of the right hand and decreased range of motion which started 1 day earlier and increased in severity. With the in-depth analysis of the patient's, he did not have any similar complaints before. An immunohistochemical examination of the biopsy material obtained from the mass on the right shoulder 1 month before the patient's numbness in his fingers was consistent with secondary amyloidosis.

In the physical examination of the patient, active and passive flexions of the fingers were found to be limited and numbness was noticed in the 1st, 2nd, 3rd, and 4th lateral half of the fingers, which is the innervation area of the median nerve. Even though no atrophy was seen in the thenar muscles, it was observed that the palm of the effected hand was hypertrophic compared to the other palm, and the Tinel and Phalen tests were both positive.

Because the direct radiographs of the patient were interpreted as normal, MRI was requested for further examination. MRI revealed excessive fluid collection and intense synovitis in the intercarpal, radioulnar, and ulnocarpal joints and surrounding para-articular soft tissue planes at the wrist level. The findings were thought to be compatible with amyloidosis, but rheumatologic diseases could also be compatible with the same findings. Because of this, further investigation has been done with clinical examination and laboratory findings. No rheumatologic diseases has been diagnosed after the investigation.

In the patient's EMG, the following are found: (1) The compound muscle action potential of the right median nerve was found to be low amplitude, and the amplitude of the muscle action potential combined with wrist stimulation decreased by more than 50 compared to palm stimulation (partial conduction block). Motor transmission speed was low in the palm-wrist segment. Motor conduction studies of the other nerves of both upper and lower extremities were found to be normal. (2) Sensory action potential of the right median nerve could not be obtained. In conclusion, these findings were consistent with acute carpal tunnel syndrome causing axon and myelin damage on the right upper extremity.

**Figure 1:**
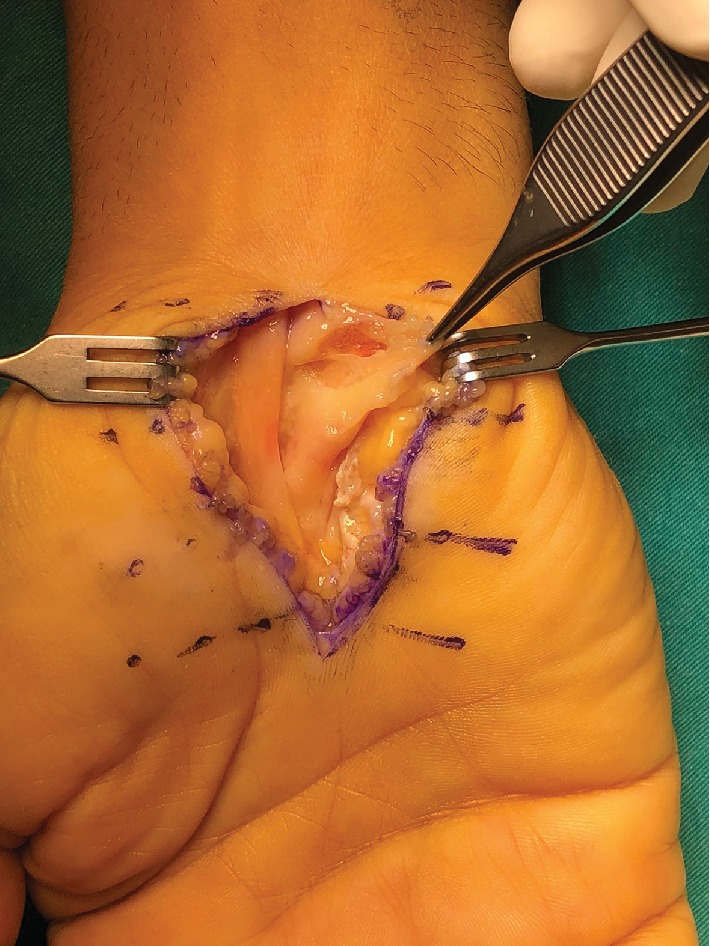
Hypertrophic synovial tissue.

**Figure 2:**
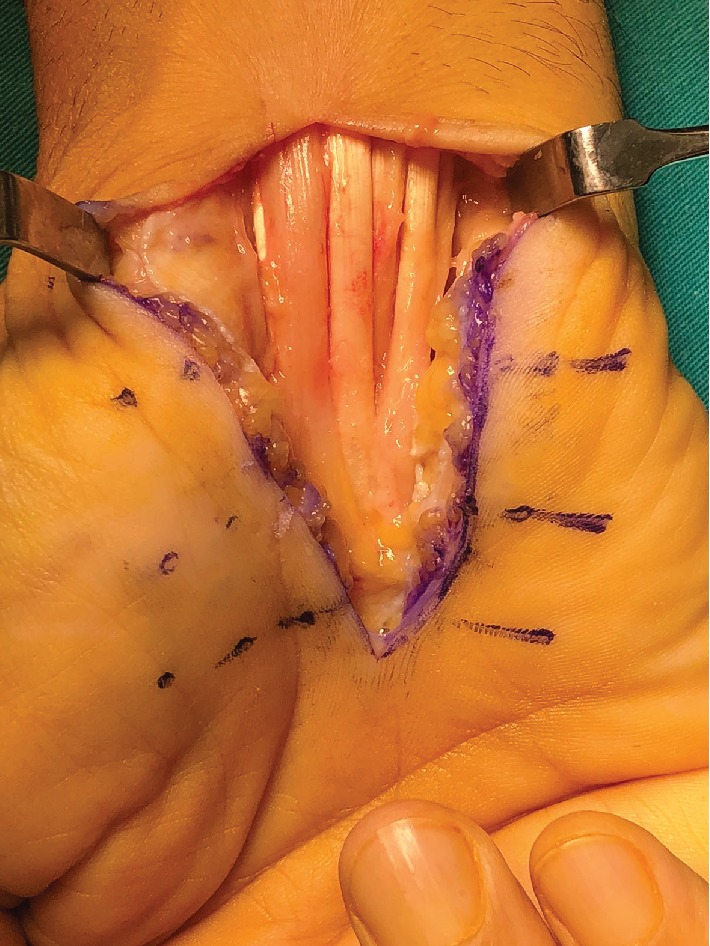
Loosened median nerve after synovectomy.

The patient underwent surgery the day after he presented to our outpatient clinic. Incision has been done in the volar side of the right hand following the palmar crisis, and the transverse carpal ligament was loosened completely; afterwards, biopsy was taken for pathology. The median nerve appear to be swelled. It was assured that the nerve was completely decompressed distally and proximally. Neurolysis was performed after the median nerve was loosened. Diffuse synovial thickening has been observed around the flexor tendons which were hypertrophic (Figure 1). Synovectomy has been performed (Figure 2).

**Figure 3:**
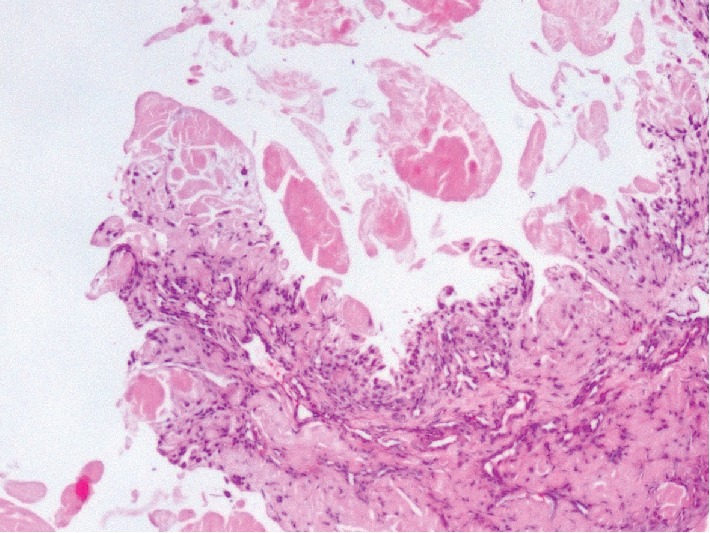
Amyloid deposits in the sample taken from the median nerve (HE stain ×100 magnification).

**Figure 4:**
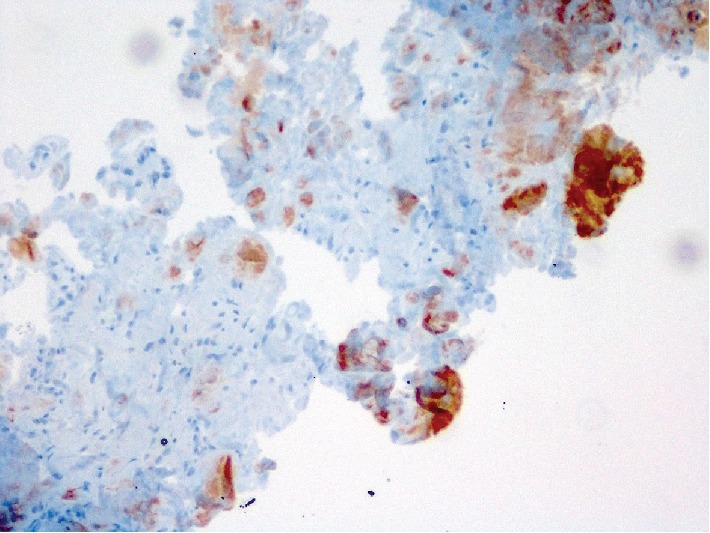
Amyloid deposits in the sample taken from the median nerve (IHC stain ×100 magnification).

According to the results of the pathology samples sent during surgery, AA-type amyloidosis was detected in the samples taken from the median nerve sheath and from the synovial tissue on the flexor tendons (Figures 3 and 4).

The Tinel and Phalen tests were found to be negative, and numbness in the fingers disappeared and finger flexion movements were more comfortable after the operation.

## 3. Discussion

The carpal tunnel, which has a constant volume of 5 ml, is surrounded by carpal bones from 3 sides and surrounded by flexor retinaculum from the volar side. The problems occur in the structures passing through this small gap further narrowing the volume, leaving a minimal space for any expansion within the carpal tunnel [2].

Carpal tunnel syndrome develops due to the prolonged and intensive use of the wrist and some systemic diseases such as rheumatologic diseases, diabetes, and amyloidosis. CTS is the most common chronic compressive neuropathy [3]. There are many studies about chronic CTS as it is a common condition. But acute CTS is a much more rare condition. CTS symptoms progress in months to years, but in acute CTS, symptoms progress very quickly in hours to days. This condition usually occurs after traumatic events [4]. Rarely, carpal tunnel syndrome may occur with atraumatic etiology such as infection, inflammation, and iatrogenic events that increase carpal tunnel pressure [4, 5].

ACTS is characterized by continuous pain and numbness along the median nerve track due to the sudden and rapid increase in pressure in the carpal tunnel. The patient complains numbness in the first 3 fingers on the affected side and in the lateral half of the 4th finger.

In the rapid process of ACTS, it is very important to make the diagnosis quickly and to plan the intervention early. The sooner the intervention is performed, the likelihood of undesirable conditions such as neural dysfunction, limitation of movement, disruption of microcirculation, and chronic pain is reduced [6].

Local amyloid deposits have been held responsible in CTS cases developing on the basis of amyloidosis, and this is not uncommon. According to Kyle et al., 1500 patients have been operated for carpal tunnel release and 124 of them had local amyloid deposits [7]. In another study, it has been shown that local amyloid deposition does not only occur in the tendons but may also occur in the transverse carpal ligament [8]. However, in these studies, no ACTS was developed on the basis of amyloidosis.

There are currently no guidelines for diagnosing ACTS or managing this process. But we know that early surgery and carpal tunnel loosening are the only ways to achieve good results. In one study, it was shown that patients who underwent surgery within 40 hours after the onset of ACTS symptoms have completely regained normal nerve function from day 4 postoperatively [9].

## 4. Conclusion

ACTS is less common than CTS. ACTS usually occurs due to trauma and begins to show serious symptoms within days to hours. Carpal tunnel decompression is always the first-line treatment in ACTS cases, whether trauma-induced or based on another etiology. As in our case, it should be remembered that acute CTS may develop while expecting chronic CTS in a previously diagnosed patient with amyloidosis.

## References

[1] Nakagawa M., Sekijima Y., Yazaki M. (2016). Carpal tunnel syndrome: a common initial symptom of systemic wild-type ATTR (ATTRwt) amyloidosis. *Amyloid*.

[2] Gillig J. D., White S. D., Rachel J. N. (2016). Acute carpal tunnel syndrome: a review of current literature. *The Orthopedic Clinics of North America*.

[3] Szabo R. M., Steinberg D. R. (1994). Nerve entrapment syndromes in the wrist. *The Journal of the American Academy of Orthopaedic Surgeons*.

[4] Kokosis G., Blueschke G., Blanton M., Levinson H., Erdmann D. (2011). Acute carpal tunnel syndrome secondary to iatrogenic hemorrhage. A case report. *Hand*.

[5] Mayne A. I., Howard A., Kent M., Banks J. (2012). Acute carpal tunnel syndrome in a patient with haemophilia. *BMJ Case Reports*.

[6] Barbee G. A., Haley C. L., Berry-Cabán C. S. (2016). A case of acute carpal tunnel syndrome. *Journal of the American Academy of Physician Assistants*.

[7] Kyle R. A., Eilers S. G., Linscheid R. L., Gaffey T. A. (1989). Amyloid localized to tenosynovium at carpal tunnel release. Natural history of 124 cases. *American Journal of Clinical Pathology*.

[8] Samões R., Taipa R., Valdrez K., Gonçalves I., Melo Pires M. (2017). Amyloid detection in the transverse carpal ligament of patients with hereditary ATTR V30M amyloidosis and carpal tunnel syndrome. *Amyloid*.

[9] Ford D. J., Ali M. S. (1986). Acute carpal tunnel syndrome. Complications of delayed decompression. *Journal of Bone and Joint Surgery British Volume*.

